# Prevalence of drug use among drivers based on mandatory, random tests in a roadside survey

**DOI:** 10.1371/journal.pone.0199302

**Published:** 2018-06-19

**Authors:** Manuela Alcañiz, Montserrat Guillen, Miguel Santolino

**Affiliations:** Department of Econometrics, Riskcenter-IREA, University of Barcelona, Barcelona, Spain; University of Toronto, CANADA

## Abstract

**Background:**

In the context of road safety, this study aims to examine the prevalence of drug use in a random sample of drivers.

**Methods:**

A stratified probabilistic sample was designed to represent vehicles circulating on non-urban roads. Random drug tests were performed during autumn 2014 on 521 drivers in Catalonia (Spain). Participation was mandatory. The prevalence of drug driving for cannabis, methamphetamines, amphetamines, cocaine, opiates and benzodiazepines was assessed.

**Results:**

The overall prevalence of drug use is 16.4% (95% CI: 13.9; 18.9) and affects primarily younger male drivers. Drug use is similarly prevalent during weekdays and on weekends, but increases with the number of occupants. The likelihood of being positive for methamphetamines is significantly higher for drivers of vans and lorries.

**Conclusions:**

Different patterns of use are detected depending on the drug considered. Preventive drug tests should not only be conducted on weekends and at night-time, and need to be reinforced for drivers of commercial vehicles. Active educational campaigns should focus on the youngest age-group of male drivers.

## Introduction

The use of drugs among the driving population is well documented as a factor associated with severe road accidents [1,2]. While there is convincing epidemiological evidence of the impairing effects of psychoactive drugs on driving [3,4,5], many studies are based on non-random samples of drivers that agree to participate in a survey [6,7] or who are recruited at leisure locations [8,9]. Studies covering the whole driving population by means of roadside surveys are scarce and drivers will typically refuse to participate [10].

The National Roadside Survey of Alcohol and Drug Use by Drivers conducted in the United States [11] found that 16% of weekend night-time drivers (roughly 1 in 6) tested positive for illicit or prescription drugs in 2007. This percentage rose to 20% in the 2013–2014 study, with the drug tetrahydrocannabinol (THC) presenting the largest increase [12]. However, this was not a mandatory survey and so its results might be biased precisely because of its voluntary nature. In the European Union (EU), the results obtained in the DRUID project, also based on roadside surveys [13,14], found that the prevalence of illicit drugs in the driving population was 1.9%, with marked differences across countries, ranging from 0.2 to 8.2%. THC (EU mean prevalence 1.32%; range: 0.00–5.99%) and cocaine (EU mean prevalence 0.42%; range: 0.00–1.45%) were the most frequently detected illicit substances in most countries. However, here again, participation was voluntary in most countries, which may lead to a substantial underestimation. Randomness in the choice of the tested driver cannot always be guaranteed completely and so, extrapolation of the results to all drivers can be challenged. In addition, the complexity of a roadside survey involving stratification, clustering and weighting is not always duly considered when presenting statistical results.

A key focal point in accident prevention is the identification of risky drivers, defined as those with an increased likelihood of driving under the influence (DUI) of drugs or alcohol. Many authors have analysed DUI in specific groups such as young adults [15] and older drivers [16]. There is also a considerable body of literature dealing with suspected drivers of drug-impaired driving [17, 18], or profiling injured or dead drivers and their risk-taking behaviour as regards substance abuse [19]. For instance, there is evidence that males injured in car crashes are more likely to test positive for alcohol and THC, whereas females who test positive are more likely to have used benzodiazepines [20]. Among fatally injured drivers, substance abuse seems to be more prevalent if the crash involves a single vehicle or occurs at night [21].

Our objective is to shed new light on road safety, by measuring the presence of drugs in the general driving population. To do this, we undertake a random sample of drivers and test them at a roadside survey, with participation being mandatory rather than voluntary. Drunk-driving is excluded as it was analysed in a previous study [22]. Our study is conducted in Catalonia, a Mediterranean region of Spain. We also seek to determine the characteristics of drivers under the influence of drugs, including their gender, age and driving patterns (day of the week and time of day, number of occupants and type of vehicle), so that we can identify risk profiles. This should help the authorities to design better educational policies and preventive campaigns, and to provide a baseline for evaluating whether significant reductions follow the implementation of enforcement interventions.

Although a few studies have been previously conducted in Spain based on mandatory drug tests [23,24,25], the novelty of our analysis lies in the combination of the mandatory nature of drug tests and the protocol to ensure randomness in the selection of drivers, in addition to the inclusion of the sample design features in the calculation of statistical results. The prevalence of drivers detected for alcohol or other substances at a police checkpoint decreases as the control time passes, probably due to the fact that drivers who go through the checkpoint warn other drivers [26,27]. Unlike previous studies in Spain, our data collection is based on the principle that only the first driver arriving at the control site is drug-tested. Hence, it is guaranteed that the selection of the driver is not influenced by factors like space availability, officers’ suspicion or time passed since the control site was set up.

## Methods

This cross-sectional study forms part of an initiative promoted by the Catalan Traffic Authority addressed at periodically measuring the prevalence of alcohol and drug-impaired driving (2014–2020 Strategic Road Safety Plan). The drivers that circulate on the region’s main interurban roads make up the population of interest. Given the impossibility of testing all drivers, a roadside survey, providing a random and representative sample, was designed. The checkpoints were randomly located across the territory divided into eight police operational areas known as Regional Traffic Areas (RTAs). Drug tests were performed by traffic officers. Participation was mandatory and with guarantees of privacy approved under the UB Riskcenter protocol (http://www.ub.edu/riskcenter/mission/). All records/information were anonymized and de-identified by the Catalan Traffic Authority prior to analysis. The ethics committee that approved this study is the UB Riskcenter Institutional Review Board with number 17UB-RK-01.

The fieldwork was conducted in the autumn of 2014. In Catalonia, spring and autumn are associated with intermediate rates of alcohol-impaired driving, whereas high peaks are typically observed in summer and significantly lower levels are found in winter [28]. The sample was collected over a month and a half, between October 1 and November 16. Days with atypical traffic flows (such as bank holidays) were intentionally excluded.

### Drug testing assessment

Spanish legislation concerning drug driving (Government Decree 1428/2003) prohibits driving following the consumption of narcotic and psychotropic drugs, as well as other stimulants or similar substances, including medicines that affect the physical or mental skills needed for safe driving. Contravening this decree is considered a serious infringement of the Spanish criminal code.

The drug tests were conducted in line with normal police procedures by traffic officers. A standardized form was used in each test to record time and location, the characteristics of the driver according to his/her driving licence (gender, age, nationality, and years holding a driving licence), type of vehicle, number of occupants, as well as the results of the test for the following substances: THC, methamphetamines, amphetamines, cocaine, opiates and benzodiazepines. This is the regular selection of substances tested by traffic officers in Catalonia. Only the first driver to pass through the checkpoint (when going into operation) was tested for drugs. The drugs test was conducted using the mobile drug screening system Alere^TM^ DDS®2 Test Kit, as a relatively wide range of illegal and prescription substances can be tested. Its cut-off concentrations are 25 ng/ml for THC, 50 ng/ml for methamphetamines and amphetamines, 30 ng/mg for cocaine, 40 ng/mg for opiates, and 20 ng/ml for benzodiazepines [29].

If the saliva sample revealed a positive result for any substance, the driver was requested to provide a larger oral fluid sample that was taken to a laboratory for confirmation. As the results of the laboratory analyses were not available for our observations, we used the outcomes of the initial saliva tests performed at the roadside. A field testing shows full agreement between the specimens from the same donors run on a roadside test for Alere DDS Test Kit and the results in the laboratory [30]. In an oral fluid drug testing study in Vermont in 2015, the overall accuracy (93.3%), specificity (96.6%) and sensitivity (70.6%) of the Alere DDS Test Kit was similar to the Dräger® Drug Test 5000 device [31].

### Stratified probabilistic sample design

A two-stage probabilistic sample was designed to gather the data. The first stage involved selecting the road sections that would constitute the primary sample units (PSUs). In a second stage, random drivers passing through these road sections formed the secondary sample units (SSUs).

The first stage of sampling required an inventory of the Catalan interurban roads. A census of the 6,910 stretches of road delimited by a given access and an exit point was provided by the traffic authorities along with their average daily traffic (ADT). Road sections with an ADT below 4,000 vehicles per day were excluded. A total of 3,469 road sections resulted eligible for selecting the PSUs by stratified sampling. The stratification variables were geographical area (RTAs), road type (conventional or motorway), flow direction (according to the road kilometre counter), day of the week, and time-slots (divided into six four-hour intervals beginning at 10 p.m.). The selection of the road sections ensured the proportional representation of RTAs and road types across the territory, as the probability of choosing a particular road section was set proportional to its length. Sampling was performed by replacement, although each road section could only be selected a maximum of three times. Half the road sections were selected in the rising flow direction and half in the decreasing direction. Given a selected road section and flow direction, the traffic agents could then choose the specific kilometre point that guaranteed the greatest safety at which to perform the tests. The selected locations were randomly equidistributed across the days of the week and time-slots.

A list of 521 selected checkpoints was provided to the police officer responsible for each RTA. The number of locations was determined by the available funding. Each designated checkpoint was supplied with a substitute location to be used if the original checkpoint could not be set up owing to unexpected factors, such as road works. That happened in 9% of the final locations. This alternative location was identical to the original one in terms of the stratification variables.

Once the selected checkpoints had been established, the second sampling stage involved selecting the individual drivers to be tested for drug use. At this stage, drivers were chosen to guarantee random sampling, so since any driver passing through the selected road section had the same probability of being chosen, the first driver approaching the checkpoint was tested for drugs, regardless of their gender, age, type of vehicle, or driving behaviour. As a result a sample of 521 drivers was obtained. The fact that only one drug test was performed per checkpoint reduces the number of resulting drug tests, but has the major advantage of eliminating the correlation between drug tests conducted in the same location in a single control site. This cluster effect could boost the standard errors and unduly cause underestimation of standard errors when ignored [32, 33].

The sample design implies that drivers circulating on a low flow road had a greater probability of being selected for the sample than those driving on roads of high traffic intensity. If we add the fact that the conventional roads were underrepresented as a consequence of the exclusion of low intensity sections, the resulting sample had to be weighted before carrying out any statistical analysis. We, therefore, weighted our drug test observations in accordance with the methodology developed in a previous study focused specifically on drunk-driving, that takes ADT into account [22].

### Statistical analysis

We recorded the characteristics of the selected drivers (Table 1), as well as the prevalence of positive results for drugs (Table 2). Table 3 shows the prevalence of the different drugs examined. Finally, a logistic regression for the presence/absence of drugs was implemented (Table 4), both for drugs as a whole, and individually for THC, methamphetamines and cocaine. All the results in Tables 2, 3 and 4 were calculated taking the complex sample design into consideration to avoid biased outcomes [32,33]. We used SAS procedure PROC SURVEYFREQ and PROC SURVEYLOGISTIC [34] to compute the prevalence results, Wald confidence intervals (CI) and logistic regressions.

**Table 1:** Sample characteristics for drivers tested for drugs.

*Characteristic*			No. of drivers	Overall, % (n = 521)	Positive, % (n = 81)
*Gender*					
	Male		462	88.7	96.3
	Female		59	11.3	3.7
*Age (in years)*					
	<25		61	11.7	16.0
	25–34		139	26.7	44.4
	35–44		171	32.8	19.8
	45–54		97	18.6	12.3
	55–64		37	7.1	7.4
	> = 65		16	3.1	0.0
*Nationality*					
	Spanish		447	86.3	88.9
	Other		71	13.7	11.1
*Full driving licence (in years)*					
	< = 2		36	6.9	10.0
	3–10		167	32.1	46.3
	11–20		153	29.4	22.5
	>20		164	31.5	21.3
*Vehicle type*					
	Van/lorry		82	15.7	24.7
	Other		439	84.3	75.3
*No*. *of occupants*					
	1		305	59.1	58.0
	2		150	29.1	29.6
	> = 3		61	11.8	12.3
*Road type*					
	Conventional		390	74.9	71.6
	Motorway		131	25.1	28.4
*Day of the week*					
	Weekend		272	52.2	49.4
	Non weekend		249	47.8	50.6
*Time*					
	6:00 a.m.–1:59 p.m.		176	33.8	37.0
	2:00 p.m.–9:59 p.m.		178	34.2	29.6
	10:00 p.m.–5:59 a.m.		167	32.1	33.3

Totals vary due to missing data. Data are unweighted.

Weekend extends from Friday 2:00 p.m. to Monday 1:59 a.m.

**Table 2:** Prevalence and 95% confidence intervals (CI) for drugs.

*Characteristic*		Prevalence for drugs. % (n = 521)	95% CI	*χ*^2^ test	*p*-value
*TOTAL*		16.4	(13.9; 18.9)		
*Gender*					
	Male	18.4	(16.0; 20.8)		
	Female	3.2	(3.1; 3.3)	9.91	0.002
*Age (in years)*					
	<25	32.9	(30.0; 35.9)		
	25–34	23.9	(20.9; 27.0)		
	35–44	10.3	(9.4; 12.1)		
	45–54	10.5	(9.8; 11.2)		
	55–64	13.4	(12.7; 14.2)		
	> = 65	0.0	-	27.79	0.000
*Nationality*					
	Spanish	16.4	(13.8; 19.1)		
	Other	17.4	(17.2; 17.6)	0.04	0.847
*Full driving licence (in years)*					
	< = 2	39.1	(30.4; 47.8)		
	3–10	21.1	(18.8; 23.4)		
	11–20	12.6	(10.5; 14.6)		
	>20	11.0	(8.6; 13.5)	18.26	0.000
*Vehicle type*					
	Van/lorry	25.6	(21.1; 30.1)		
	Other	14.2	(11.7; 16.8)	7.65	0.006
*No*. *of occupants*					
	1	14.5	(12.7; 16.3)		
	2	19.3	(17.4; 21.2)		
	> = 3	22.5	(19.7; 25.2)	2.98	0.226
*Road type*					
	Conventional	15.6	(12.8; 18.4)		
	Motorway	17.6	(12.8; 22.4)	0.37	0.544
*Day of the week*					
	Weekend	13.3	(10.5; 16.1)		
	Non weekend	18.7	(14.8; 22.6)	2.69	0.101
*Time*					
	6:00 a.m.–1:59 p.m.	15.5	(12.0; 19.0)		
	2:00 p.m.–9:59 p.m.	14.6	(12.2; 17.1)		
	10:00 p.m.–5:59 a.m.	21.5	(12.3; 30.7)	2.74	0.254

Totals vary due to missing data.

Weekend extends from Friday 2:00 p.m. to Monday 1:59 a.m.

**Table 3:** Prevalence and 95% confidence intervals (CI) for specific drugs and their combinations.

	Prevalence of each drug					
*Drug*	Overall %	95% CI*	As a single drug	95% CI	Combined with other drugs	95% CI
THC	12.4	(10.2; 14.7)	10.5	(8.3; 12.8)	1.9	(1.5; 2.3)
Methamphetamine	3.4	(2.5; 4.4)	1.0	(0.1; 1.9)	2.4	(1.7; 3.1)
Amphetamines	2.2	(2.1; 2.4)	0.0	-	2.2	(2.1; 2.4)
Cocaine	1.8	(0.9; 2.8)	0.7	(0.3; 1.1)	1.1	(0.3; 2.0)
Opiates	0.7	(0.0; 1.4)	0.4	(0.0; 1.1)	0.2	(0.0; 0.7)
Benzodiazepines	0.4	(0.1; 0.7)	0.0	-	0.4	(0.1; 0.7)
TOTAL	16.4	(13.9; 18.9)	12.7	(10.3; 15.1)	3.7	(2.8; 4.7)

Intervals may not be symmetric due to rounding effect.

**Table 4:** Logistic regression model for the presence/absence of any substance, THC, methamphetamine or cocaine.

	Any substance	THC	Methamphetamine	Cocaine
	OR (95% CI)	OR (95% CI)	OR (95% CI)	OR (95% CI)
Gender (ref. female)				
Male	7.00 (4.45; 11.03)**	5.89 (3.70; 9.37)**	- (^a^)	7.15 (2.78; 18.36)**
Age (years)	0.96 (0.93; 0.98)**	0.95 (0.93; 0.98)**	0.99 (0.93; 1.07)	0.97 (0.93; 1.01)
Vehicle type (ref. Other)				
Van/lorry	2.34 (1.28; 4.28)**	1.32 (0.76; 2.27)	4.28 (1.17; 15.58)*	2.74 (0.89; 8.49)
No. of occupants	1.30 (1.01; 1.66)*	1.20 (0.93; 1.55)	1.80 (1.17; 2.76)**	0.47 (0.17; 1.30)
Time (ref. 6:00 a.m-9:59 p.m.)				
10:00 p.m.–5:59 a.m.	1.66 (0.89; 3.07)	1.53 (0.78; 3.00)	2.75 (1.14; 6.65)*	2.93 (0.83; 10.41)
*χ*^2^ H-L	10.63 (0.22)	8.22 (0.41)	6.33 (0.61)	3.60 (0.89)

OR = odds ratio estimate; CI = confidence interval; *χ*^2^ H-L = Hosmer-Lemeshow test (p-value); Ref. = reference category.

^(a)^ All positive tests were men.

Significance level at 1% (**) and 5% (*).

The classification variables were gender, age, nationality, years holding a driving licence (note that the minimum legal age for driving in Spain is 18 years), vehicle type, number of occupants, road type, day of the week, and time of day. The categorization of these variables was limited by the relatively small sample size. Consequently, age was categorized into six intervals covering young and inexperienced drivers (aged less than 25 years), four 10-year intervals, and old drivers (aged 65 years or more). Nationality was categorized as Spanish or other. As suggested elsewhere [35], vehicle type was categorized into van or lorry and other. In this way, we could analyse the differences between vehicles usually driven for professional purposes and others (mainly cars, motorbikes and mopeds). The number of occupants, including the driver, was defined as 1, 2 or 3+, as there were few cases with 4 or more occupants. Following the sample design, the roads were classified into two groups: conventional or high-speed (motorways). In the case of the days of the week, we discriminated between the weekend (from Friday 2:00 p.m. to Monday 1:59 a.m.) and weekdays. In order to have a sufficiently large sample size for the weekend, Tuesday to Thursday was considered as presenting homogeneous traffic behaviour and treated as a single day in the sample stratification; for the prevalence calculation, however, these observations were properly weighted to represent three separate days. The six time-slots considered in the sample design were grouped into three intervals for the analysis: 6:00 a.m. to 1:59 p.m. (morning), 2:00 p.m. to 9:59 p.m. (afternoon/evening), and 10:00 p.m. to 5:59 a.m. (night).

## Results

### Characteristics of tested drivers

Catalonia had 7.5 million inhabitants in 2015 (49.1% men, 50.9% women), and 26.2% were under 40 years of age. Official figures indicate that 4.2 million citizens in Catalonia (Spain) have a driving licence and from those 57.9% are men and 42.1% are women. That is, many more men than women have a driver’s licence.

The features of the driver, the vehicle and the road type, along with the day and time-slot in which the test was performed are shown in Table 1 (unweighted statistics), which also reports the distribution of the 81 drivers that tested positive. Drug-tested drivers were more likely to be male (88.7%), aged 25–44 years (59.5%), Spaniards (86.3%) and to have held a driving licence for 3 to 10 years (32.1%).

As in other studies in Spain [23,24], the majority of drug-tested drivers were males. Those studies report that 79.9% and 81.5% of drug-tested drivers were males. We obtain a larger percentage, which can be due to the fact that our analysis is focused only on interurban roads. Previous studies have observed that there is a substantial difference in Spain between the average kilometres per day in men (34.0 km/day) and women (28.1 km/day) [36]. This means that women not only hold a driving licence less frequently than men but they also drive shorter distances on average. This makes women much less likely to be stopped in a roadside random survey than men.

As expected, the linear correlation between the driver’s age and the years since he gained a driving licence was positive and strong (Pearson’s linear correlation coefficient = 0.81). Vehicles stopped for testing were mainly for private use (not van or lorry, 84.3%) and had one occupant (74.9%). Tests were most frequently carried out on conventional roads (74.9%), during the weekend (52.2%) and in the time-slot between 2:00 p.m. and 9:59 p.m. (34.2%).

Positive outcomes for drugs were over-represented for males (96.3%) and drivers aged 25–34 years (44.4%). The same was true for Spaniards (88.9%) and drivers holding a licence for a maximum of 2 years (10.0%) or for 3–10 years (46.3%). Positives for drugs were also over the expected values for van and lorry drivers (24.7%), circulating on a motorway (28.4%), not on the weekend (50.6%) and during the morning (37.0%).

### Prevalence of drug use

As shown in Table 2, 16.4% of the tested drivers were positive for at least one drug (95% CI: 13.9–18.9%). Male drivers had significantly higher prevalence rates of drug use than female drivers: 18.4% of males (95% CI: 16.0–20.8%) vs. 3.2% of females (95% CI: 3.1–3.3%). Young drivers were more likely to test positive. The highest prevalence of drug use was detected in drivers under the age of 25 years (32.9%, 95% CI: 30.0–35.9%) and between the ages of 25 and 34 years (23.9%, 95% CI: 20.9–27.0%). Middle- aged drivers had lower rates of drug driving than average: 10.3% (95% CI: 9.4–12.1%) for drivers aged 35–44 years and 10.5% (95% CI: 9.8–11.2%) for those aged 45–54 years. A slightly higher prevalence was found for drivers aged 55–64 years (13.4%, 95% CI: 12.7–14.2%), while no cases were detected among those aged 65 years or more. As for the relationship between drug use and driving experience, the highest prevalence of drug use was detected in drivers holding a driving licence for a maximum of 2 years (39.1%, 95% CI: 30.4–47.8%).

Drivers of vans or lorries tested positive for drug use more frequently than drivers of other vehicles (25.6%, 95% CI: 21.1–30.1%, and 14.2%, 95% CI: 11.7–16.8%, respectively). Finally, in terms of vehicle occupation, the lowest prevalence was found when the vehicle had just one occupant (14.5%; 95% CI: 12.7–16.3%). Nevertheless, the *χ*^2^ test for the association between the number of occupants and the presence of drugs was not statistically significant at the 5% significance level (*χ*^2^ = 2.98, df = 2, p-value = 0.226). Similarly, the test was not statistically significant at the 5% significance level if vehicles with two or more occupants were classified together and compared with vehicles with one occupant (*χ*^2^ = 2.714, df = 1, p-value = 0.099).

The rest of the characteristics analysed in order to detect differences in the prevalence of drug driving were not statistically significant. Thus, no differences were detected between Spanish and foreign drivers. The type of road on which the checkpoint was located was also not significant. The prevalence of drug detection did not statistically differ between the weekend and the rest of the week and, similarly, the time interval in which the tests were performed did not show any significant differences in positive outcomes. We also compared the drug use among drivers during daytime hours (6:00 a.m.-9:59 p.m.) and night-time hours (10:00 p.m.-5:59 a.m.) (*χ*^2^ = 2.68, df = 1, *p*-value = 0.102) and no significant difference is obtained at the 95% confidence level.

### Drug category and combinations

Among the drivers tested for drugs, THC was the most commonly detected drug (12.4%, 95% CI: 10.2–14.7%), followed at some distance by methamphetamines (3.4%, 95% CI: 2.5–4.4%) and amphetamines (2.2%, 95% CI: 2.1–2.4%). The other drugs detected were cocaine (1.8%, 95% CI: 0.9–2.8%), opiates (0.7%, 95% CI: 0.0–1.4%) and benzodiazepines (0.4%, 95% CI: 0.1–0.7%) (Table 3).

If we focus on poly-drug use, 3.7% of drivers tested positive for multiple drugs (95% CI: 2.8–4.7%). THC was detected as a single drug in 10.5% of drivers (95% CI: 8.3–12.8%), while its prevalence in combination with other drugs was 1.9% (95% CI: 1.5–2.3%). All drivers testing positive for amphetamines tested positive for at least one other drug, primarily methamphetamines (1.8%, 95% CI: 1.7–1.9%). Benzodiazepines were also detected in combination with other drugs in all cases (0.4%, 95% CI: 0.1–0.7%). For methamphetamines, cocaine and opiates, no differences were detected in the prevalence of the single use of these drugs or their combined use with other drugs.

### Logistic regression analysis

Multivariate analysis aimed to consider interactions between variables was carried out by means of a logistic regression. Aiming to improve the logistic regression analysis, the driver’s age and the number of occupants were directly included as count variables. Age ranged from 16 to 78 years, and the vehicle occupation was between 1 and 5 individuals. Time-slots of day-time hours were combined (6:00 a.m.–9:59 p.m.) and compared with night-time hours (10:00 p.m.–5:59 a.m.).

Variables affecting the likelihood of testing positive for any drug or, separately, for THC, methamphetamine or cocaine are shown in Table 4. In all the cases the Hosmer-Lemeshow (H-L) test indicated a good goodness of fit of the logistic regression. The regression analysis was also performed for amphetamines and the H-L statistic was significant at 1% indicating a poor goodness of fit (*χ*^2^ H-L, p-value<0.01). The individual regression analysis for the rest of drugs is not reported due to the low number of positive outcomes.

Logistic regression analysis showed that the likelihood of finding a driver positive for any substance was higher among men (OR = 7.00, 95% CI = 4.45–11.03), among drivers of vans or lorries (OR = 2.34, 95% CI = 1.28–4.28), increased with the number of occupants (OR = 1.30; 95% CI = 1.01–1.66), and decreased with age (OR = 0.96, 95% CI = 0.93–0.98). The parameter for night-time hours (10:00 p.m.-5:59 a.m.) was not significant at the 5% significance level when the category of reference was day-time hours (6:00 a.m.-9:59 p.m.) (OR = 1.66, 95% CI = 0.89–3.07, p-value = 0.076).

The likelihood of detecting a driver positive for THC was associated with male gender (OR = 5.89, 95% CI = 3.70–9.37) and age (OR = 0.95, 95% CI = 0.93–0.98), whereas methamphetamine positive results were associated with driving vans or lorries (OR = 4.28, 95% CI = 1.17–15.58), the number of occupants (OR = 1.80, 95% CI = 1.17–2.76) and night-time (OR = 2.75, 95% CI = 1.14–6.65). When the use of cocaine was analysed, the risk of testing positive was mainly associated with males (OR = 7.15, 95% CI = 2.78–18.36). Coefficients associated with younger individuals (OR = 0.97, 95% CI = 0.93–1.01, p-value = 0.088), driving vans or lorries (OR = 2.74, 95% CI = 0.89–8.49, p-value = 0.080), and at night (OR = 2.93, 95% CI = 0.83–10.41, p-value = 0.095) were not statistically significant at the 5% significance level.

## Discussion

Drunk-driving interventions seem to have been effective in cutting alcohol prevalence rates [37,38]. However, corresponding rates of driving under the influence of non-alcohol drugs and their consequences on road fatalities continue to show a disturbing upward trend [21,39]. Our findings reveal that the prevalence of non-alcohol drugs is as high as 16.4% in Catalonia, notably higher than the prevalence of alcohol impaired-driving, estimated at 1.3% [22]. Clearly, drug use constitutes a public health issue and our results point to a complex problem.

Although achieving a drug-free world is an unrealistic goal, the fight to reduce drug-associated dangers, such as drugged driving, needs to be given priority status. Public policies, focused on education and prevention, are necessary to raise awareness of the hazards of substance abuse and its consequences for road safety. Here, the determination of the socio-demographic characteristics of those groups at greatest risk of drug driving is crucial. In line with previous studies [40,41], our results show that male drivers are more likely to test positive for drugs than females. However, an examination of recent trends in drunk-driving indicates that the gender gap is narrowing in terms of the number of DUI arrests [42].

A driver’s age is a highly significant factor explaining the likelihood of driving under the influence of drugs, with young drivers being more likely to offend than older drivers. Being in possession of a full driving licence for less than two years is also a highly relevant risk factor, indicating that inexperienced, young (male) drivers constitute the group at greatest risk. This profile, however, may not be the same in the case of drunk-driving, with some studies reporting that middle-aged drivers are more likely to test positive for alcohol than younger drivers [26], while others continue to cite the higher prevalence of alcohol-impaired driving among younger demographic groups [43,44].

Our results also point to the disturbing use of methamphetamines among drivers of vans and lorries. Previous research has, likewise, identified a higher prevalence of methamphetamines and other stimulants among commercial drivers [45]. Driving with passengers is also positively associated with drug use, especially methamphetamines. This result coincides with earlier studies that show that drug use is related to the number and composition of occupants in a vehicle [46]. These findings should be helpful in understanding the social use of certain drug types and the behavioural nature of driving under their influence.

Consistent with other roadside surveys, we did not find drugged driving to be especially concentrated on weekends [23, 46]. However, we found methamphetamine use among drivers to be associated with night-time hours, although THC and cocaine use is not associated with a specific time-slot. Earlier studies suggest that drugged driving, in contrast to drunk-driving, is less concentrated in terms of the day of week and time of day [12]. Overall, our results suggest that different patterns of drug use among drivers emerge depending on the type of drug. These conclusions may be useful for police officers, who need to identify the signs and symptoms of drug use and to have an appreciation of patterns in prevalence variation.

As in other studies [47, 48], THC is by far the most prevalent drug identified in our analysis. Studies indicate that the frequency of cannabis use in the population declines with age, with young people most likely to start using cannabis and to use it on a regular basis throughout their youth [49,50]. Although the cannabis derivatives of marijuana and hashish are often considered soft drugs, causing fewer health problems than other drugs [51], there is evidence that THC use increases the risk of collision [2,5]. The current debate concerning cannabis legalization in various countries needs to take into account that, if implemented, there may be an increase in the prevalence of THC use by drivers [48,52], and hence a negative impact on road safety.

The particular strengths of the current study, compared to previous drug driving studies, are: (i) it is based on a stratified probabilistic sample specifically designed to be representative of all circulating vehicles; (ii) the selection of the driver to be drug-tested is not influenced by such factors as availability of space, officers’ suspicions or time elapsed since the control site was set up; (iii) the drug test is compulsory for all drivers stopped, which eliminates any potential respondent bias that might emerge when participation is voluntary, and (iv) risk factors that are rarely studied, such as the number of occupants in the vehicle, are analysed here.

However, the study is not without its limitations. For example, no time data were available, so trends over time could not be studied. Data were collected in autumn, following the alcohol-use seasonality pattern presented by drivers on Catalan roads [28]. However, drug-use seasonality may differ, which means our results might only reflect drug prevalence in autumn. Additionally, caution should be exercised when a high degree of disaggregation (joint analysis of multiple characteristics) is undertaken given the sample size, particularly when differences in the types of drug consumed are studied. Another limitation is that drivers with drug concentrations below cut-off levels could not be identified and, in the case of cannabis, the THC cut-off level used by Spanish police [53] may differ from the benchmarks employed in other jurisdictions [54]. A further restriction is that the presence of drugs was tested using oral fluid samples, given that blood sample results from laboratory analyses were unavailable. We should also note the lack of information about medicines other than benzodiazepines, including herbal medicines, taken by patients (prescription and/or over-the counter), and also drug-drug and drug-alcohol interactions that could modify levels of detected drugs.

Drunk-driving behaviour on Catalan roads, where a breath test is considered positive if the breath alcohol concentration (BrAC) is >0.25 mg/L or, equivalently, the blood alcohol concentration (BAC) is >0.5 g/L, has been previously studied following a similar sample design methodology to the one used here [22,55]. Indeed, we recognise that this study would be more informative if a comparison with the prevalence of alcohol use was also provided. Unfortunately, not all drivers tested for drug use were simultaneously tested for alcohol. In the subsample where drug and alcohol test outcomes were available for the same driver, we found that 16.5% of the drivers tested positive only for drugs, 1.5% only for alcohol, and 0.1% of the cases tested positive both for alcohol and drugs. Given the high percentage of missing data, we opted to exclude alcohol from the analysis. Although considerable caution is required, our findings would seem to be in line with previous studies that report that the combined use of alcohol and drugs is less frequent than their separate use [23,24,53].

The alarming prevalence of drugged driving reported here suggests that the consumption of illegal substances is widespread in Catalonia, especially among the young male population. As such, this is a significant public health issue that needs to be addressed via education, prevention and enforcement. Promoting a more complete understanding of the risks involved in alcohol- and drug-impaired driving is critical for reducing fatalities on our roads. The use of drugs among the Catalan population has not been shown to differ from that in other Spanish regions, so these results could likely be extrapolated to other parts of the country; however, greater caution should be exercised when extrapolating them to other countries.
